# Foreign Gleanings

**Published:** 1874-06-01

**Authors:** F. J. Huse


### FOREIGN GLEANINGS.

COLLATED BY F. J. HUSE, M.D.

CROTON-CHLORAL.--By treating aldehyde with chlorine a new product is formed, which, although it has not, as might at first be supposed from its name, any relation to croton-oil, has been named croton-chloral. From ordinary chloral it is distinguished by its much readier solution in water, its crystallization in small brilliant flakes, and especially by its physiological properties.

A dram introduced into the stomach, in an aqueous solution, produced profound sleep with anesthesia in twenty minutes. Moreover, while the cutaneous sensibility is destroyed, the muscular tonicity remains without any alteration, so that the discoverer has seen several instances where patients subjected to its influence have remained in a sitting posture without falling. In addition, there is neither any modification of the respiration nor of the pulse.

In certain cases of neuralgia one can mark the subsidence of the pain previous to the appearance of sleep. Consequently the discoverer claims special advantages in the employment of this remedy in many cases where one would make use of large doses of either chloral or opium.

Wickman Legg has made use of this medicine in twenty cases of neuralgia of various degrees of intensity and duration, in doses of from five to fifteen grains in the form of an aqueous solution. The results were highly satisfactory. In only two cases was it unsuccessful. In all the others it caused an entire disappearance of the pain.

Benson Baker reports five cases of long standing and very painful neuralgia which were entirely cured or, at the least, greatly improved. The results were the same in unusual neuralgic pains of the face. Moreover the remedy neither produces vomiting nor headache. — *Schmidts Jahrbuch*, 1874, *No.* 1, *p.* 17.

---

SUBCUTANEOUS INJECTION OF CARBOLIC ACID IN ACUTE ARTICULAR RHEUMATISM — In confirmation of the teachings of Hüter that carbolic acid should prove one of the best autiphlogistics, we note, in the *Deutsche Zeitschrift*, the clinical history of four cases of rheumatic fever with severe arthritis, in which Dr. Kunse has made use of carbolic acid with highly favorable results. One of these patients who had been suffering for over two weeks, and who presented the conditions of a pulse of 120, hot skin, great thirst, urine highly charged, and intensely painful knees, was subjected to a subcutaneous injection on the external aspect of the left knee from a Pravaz syringe charged with a solution of one part of carbolic acid to one hundred of water. The relief following this administration was so great that the patient demanded next morning an injection for the other side. In addition to the lessening of the pain, it produced a subsidence of the febrile action, diminution of the pulse, and sleep. In the three other cases it was attended by the same success.

